# Failure Mechanism of Hybrid Steel Beams with Trapezoidal Corrugated-Web Non-Welded Inclined Folds

**DOI:** 10.3390/ma14061424

**Published:** 2021-03-15

**Authors:** Ahmed S. Elamary, Yasir Alharthi, Osama Abdalla, Muwaffaq Alqurashi, Ibrahim A. Sharaky

**Affiliations:** Civil Engineering Department, College of Engineering, Taif University, P.O. Box 11099, Taif 21944, Saudi Arabia; y.harthi@tu.edu.sa (Y.A.); omohamed@tu.edu.sa (O.A.); m.gourashi@tu.edu.sa (M.A.); i.sharaky@tu.edu.sa (I.A.S.)

**Keywords:** corrugated webs, failure mechanism, hybrid steel beams, flange stiffener, flexural behaviour

## Abstract

Literature of Steel Beams with a thin-walled trapezoidal Corrugated Web (SBCWs) shows that the capacity of SBCWs is affected by both the fatigue cracks initiated along the inclined folds (IFs) and the maximal additional stress located in the middle of the IFs. An experimental investigation on the behaviour of hybrid SBCWs under flexure is presented in this paper. This study focuses on the effect of the welding IF between the web and flanges (IFs welded or non-welded), the horizontal-fold length (200, 260, and 350 mm), and transversal flange stiffeners on the failure mechanism of the SBCW under three line load. Accordingly, six hybrid specimens were fabricated, instrumented, and tested (five SBCW specimens and one specimen with a flat web). The test setup was designed to generate shear and a moment in the testing zone via three-point bending. The results indicated that non-welded IFs specimens with or without flange stiffeners failed owing to web tearing after web and flange local buckling. The failure mode of the specimen with continuous welding between the web and flanges was local flange buckling. Finally, the paper presents a comparison between the experimental results and the European Code to predict the capacity of the flange towards local buckling. It was concluded that the non-welding the IFs affected the inelastic behaviour and the capacity of the SBCWs. In addition, the bending resistance equations presented by EN 1993-1-5 can safely predict the test results of the non-welded inclined fold and yield a high safe variation.

## 1. Introduction

Using high-strength steels in building construction is essential, as it reduces not only the carbon dioxide emissions in the steel industry but also the total steel consumption compared with the use of conventional steel. It is well known that the failure mechanism of steel beams with trapezoidal corrugated webs (SBCW) develops in two stages: web buckling (controlled by the shear stress) and flange buckling (controlled by the yield stress). The standards and theories presume that the shear buckling stress ranges from 55% to 60% of the material yield strength. AISC [1] restricted the web slenderness ratio to prevent web shear buckling before flange yielding (buckling). The hybrid SBCW is a special type of beam with different web and flange yield stresses. A hybrid section with a web yield stress higher than the flange yield stress is appropriate for achieving web and flange buckling at approximately the same time. A study conducted by Kengo Anami et al. [2] revealed that the fatigue cracks were initiated along the inclined folds (IFs) and then spread perpendicular to the principal stress direction, affecting the beam capacity. Thus, in the present study, the effect of non-welded IFs on the behaviour of SBCWs was investigated by eliminating the possibility of failure due to the propagation of cracks initiated near the ends of the IFs. A few previous studies focused on hybrid SBCWs or non-welded IFs; therefore, general background information regarding the behaviour of SBCWs (shear and bending)—including the hybrid section and welding—is presented below.

The corrugated webs are composed of horizontal folds (HFs) and IFs supporting each other in accordance with the folded plate theory. Owing to the support provided by each panel to its neighbouring panels (i.e., the corrugated web panel behaves as a vertical stiffener to the other panels), the out-of-plane stiffness of a corrugated web is higher than that of a plane steel web. Thus, the number of stiffeners and the web thickness can reduce the steel consumption. For SBCWs, owing to the accordion effect of the web panels, the bending moment is assumed to be carried entirely by the flanges, and the web carries almost the entire shear force with a uniform stress distribution over the web height [3]. Some studies have focused on SBCWs owing to their superb characteristics; most of these reports dispensed with the shear and bending responses of simply supported beams.

First, regarding the shear response, few experimental and theoretical investigations on the shear buckling appearance and shear strength of trapezoidal corrugated steel webs (CSWs) have been conducted. For instance, the CSW is expected to enhance the shear ability of steel I beams (SIBs), as the shear yielding and buckling of the web govern the shear strength of a SIB [3,4,5]. The buckling of trapezoidal corrugated panels under in-plane loading using the spline finite strip method and finite-element (FE) method was analysed [5], and accordingly empirical formulae were proposed for predicting the shear capacity of CSW girders. As for SBCWs, Shimada S. [6] has investigated the shear response of SBCWs since 1965. Elgaaly et al. [7] and others [8,9,10,11,12,13,14,15,16,17] conducted analytical and experimental studies on SBCWs loaded mainly with shear force. Subsequently, the shear strength and flexural response of SBCWs were further examined [18,19,20,21,22,23,24]. This research, mostly, was concerned with developing formulae for predicting the local and global shear buckling and the interaction between the two shear buckling phenomena, along with the corresponding theory. Furthermore, researchers have studied many factors that may affect the shear strength of trapezoidal corrugated-web beams, such as the initial geometric imperfections, interaction between local and global shear, web slenderness, corrugation density, web thickness, panel width, web height, and steel grade. These studies revealed that shear stress is maximized and uniformly distributed throughout the web until buckling, and stocky corrugated webs were shown to reach the shear yield strength. Furthermore, they observed local web buckling in coarse corrugations and global buckling in dense corrugations. Lindner and Aschinger [25] suggested using 70% of the shear buckling stress as the nominal shear strength to design a SBCW.

Second, regarding the bending response, quite a few previous investigations focused on the determination of the bending resistance of composite SBCWs [26,27,28]. These investigations indicated that there is no relationship between the flexural and shear behaviours of SBCWs. However, the flange yield strength exhibited a precarious impact on the moment capacity of steel beams with CSWs. In the case of a SBCW subjected to an in-plane moment and shear, Abbas et al. [19] reported that a corrugated web I-girder under an in-plane moment and shear was not only deflected in-plane but also twisted out-of-plane simultaneously. Flange transverse bending produces flange transverse displacements and flange stresses that add to the stresses arising from in-plane bending. Abbas et al. [20] introduced a simplified analytical method referred to as the C-factor method, which considerably simplifies the calculation of the flange transverse bending. The flange transverse bending of corrugated web I-girders under in-plane loads was first observed and studied in Germany by Lindner et al. [29,30]. However, corrugated-web beams have weaknesses owing to their geometric characteristics. First, the local buckling strength of the flange can be smaller than that of a flat-web beam because the outstand of the flange in beams with corrugated webs is larger than that in beams with flat webs [31]. Second, because of web eccentricity, an additional in-plane transversal moment occurs in the flanges [18]. This moment reduces the flexural strength of the corrugated-web beam. Third, with the exception of the web, only the flanges contribute to the flexural strength of corrugated-web beams, owing to the accordion effect of the corrugated webs [24].

Regarding welding research, few investigations have been performed on the welding between the web and the flanges, which were first studied by Sherman and Fisher [32]. They tested 25 SBCWs with three distinctive web thicknesses to determine the number of connections needed between the flanges and the web for achieving the full material strength. It was found that only the HFs needed to be connected to the flanges. Additionally, the connection between the IFs and the flanges had negligible effects on the beam strength and stiffness. The numerical model presented by Kollar and Kovesdi [33] can determine the specific imperfection shape and residual stresses for a corrugated web girder geometry and manufacturing method. They presented advanced models capable of simulating the realistic behaviour of girders and recommended verification via laboratory tests. Kollar and Kovesdi [34] also developed a FE model for the thermal cutting and welding of corrugated web girders, which can simulate thermal phenomena during manufacturing and is useful for determining the residual stresses and initial geometric imperfections according to the welding variables. The results indicated that in the flanges of corrugated web girders, there is a significant transverse bending moment due to manufacturing, which increases the compressive residual stresses. The magnitudes of the tensile residual stresses within the flange and web of a corrugated web girder are similar to those for flat web girders.

The yielding stresses of the flanges and web significantly affect the buckling behaviour of steel beams with corrugated webs (SBCWs). The hybrid SBCW is a special type of beam with different yield stresses for the web and flanges. Knowledge regarding the flexural behaviour of hybrid SBCWs is limited—despite their importance—owing to the lack of research on this problem. Studies have demonstrated the need for further investigations, particularly in the case of non-welding the IFs between the web and flanges.

## 2. Research Objectives

In the present study, the flexural behaviour of a hybrid SBCW non-welded inclined fold was experimentally examined. The study focused on three parameters that may affect the flange or web buckling phenomena: non-welding IFs between the web and the flanges, the HF length, and the flange transversal stiffeners. Five hybrid SBCWs and one steel beam with a flat web were instrumented and tested under three-line loads. Additionally, the flange or web buckling phenomena were compared between a trapezoidal corrugated-web beam and an alternative beam with a flat web. The main objectives of this study were as follows:

1. To test a trapezoidal SBCW hybrid section until failure under in-plane bending and shear
2. To investigate the performance of a hybrid SBCW with various HF lengths under bending and constant shear force throughout the beam length
3. To experimentally determine whether the non-welding of the IFs can affect the failure mechanism and capacity of hybrid SBCWs
4. Eliminating the failure, this might exist because of the flange local buckling, by using a very limited number of stiffeners in a certain place

## 3. Experimental Programme

The experimental work described in this section was part of a research programme conducted to identify factors that may affect the flexural behaviour and strength of hybrid SBCWs.

### 3.1. Fabrication and Details of Specimens

**Figure 1:**
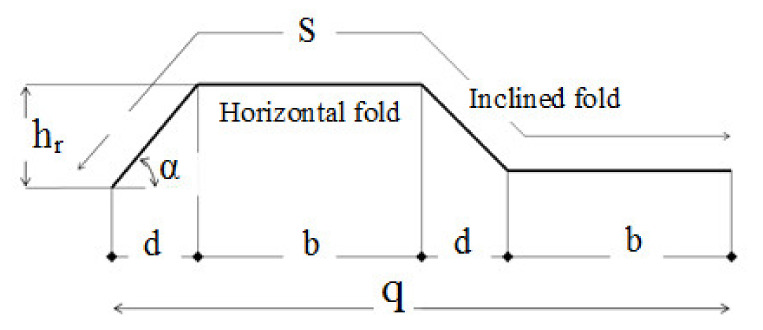
Corrugated web: profile configuration.

Five full-scale SBCWs and one flat-web beam were tested under three-line loads. The tested beams had an approximate length of 1900 mm and an effective span of 1800 mm. The web height (*h_w_*), web thickness (*t_w_*), flange width (*b_f_*), and flange thickness (*t_f_*) were 400, 3, 200, and 8.0 mm, respectively. The height-to-thickness ratio of the corrugated web (*h_w_/t_w_*) was 133. Because the flange compactness measured for a corrugated-web beam (i.e., the outstanding length) was variable throughout the inclined panel length, the compactness of the flanges in the tested specimens was measured with respect to the maximum outstanding length ((*h_r_ + b_f_*)/2*t_f_*). Three different HF lengths (200, 260, and 350 mm) were examined. The corrugation depth (h) and the horizontal projected length of the IF (d) were equal to 100 mm. The corrugation angle (α) was 45°. The corrugation profiles of the specimens are shown in Figure 1.

For each specimen, three steel-plate stiffeners (400 mm, 200 mm and 8 mm) were used. There was one over each support, and the third was under the concentrated load. The corrugated web was connected to the flanges with continuous 4-mm fillet welds from one side with a connection degree of 45° using gas metal arc welding. The size of the weld for connecting the built-up section and the end connecting plates was in accordance with EN 1993-1-5 [35]. Welding safety procedures were followed to avoid distortion of the beam resulting from the high temperature of the welding process, particularly for slender parts. Each specimen was identified by a code denoting the tested parameters, where ‘CW’, ‘FW’, ‘IF’, ‘FS’, ‘W’, and ‘NW’ represent ‘corrugated web’, ‘flat web’, ‘inclined fold’, ‘flange stiffeners’, ‘welded’, and ‘non-welded’, respectively. The number following ‘CW’ or ‘FW’ indicates the HF or intermittent welding line length (in cm), respectively. The details of each specimen are presented in Table 1.

**Figure 2:**
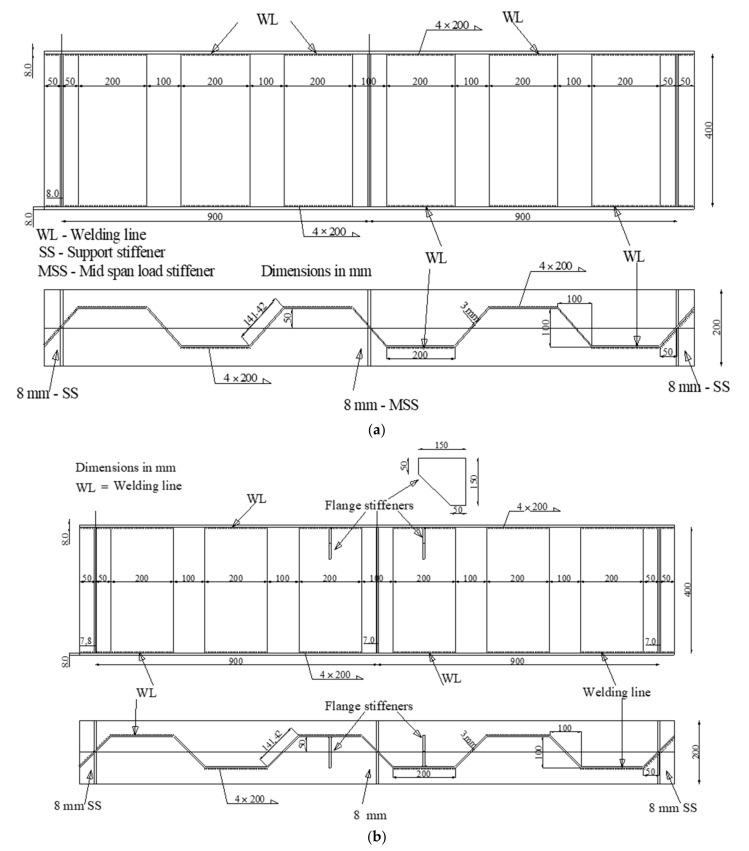
Details of the tested beams with a horizontal fold (HF) length of 200 mm: (**a**) Specimen CW20IFNW; (**b**) Specimen CW20IFNWFS.

**Figure 3:**
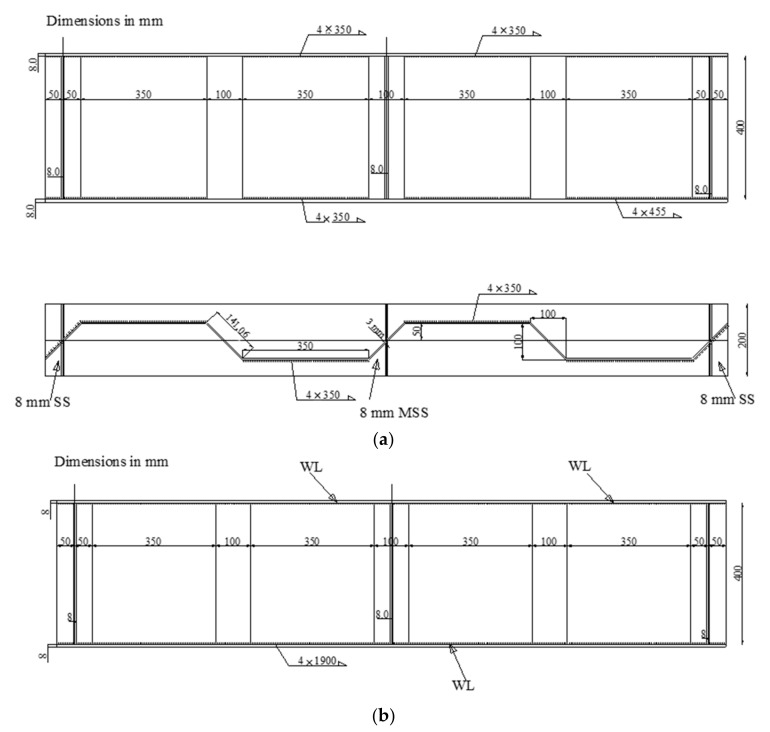
Details of the tested beams with a HF length of 350 mm: (**a**) CW35IFNW; (**b**) CW35IFWL.

**Figure 4:**
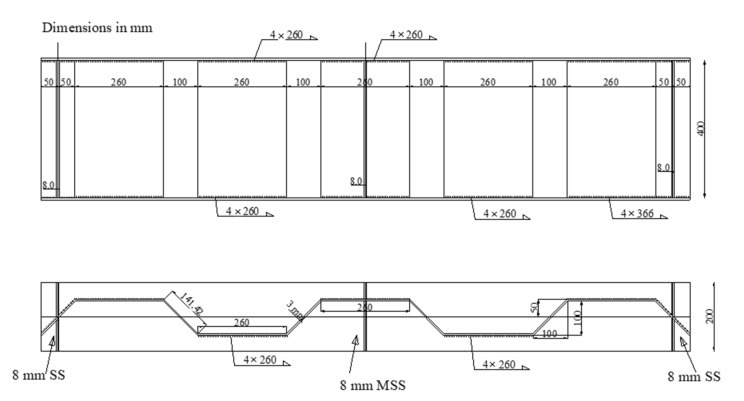
Details of the corrugated-web specimens (CW26IFNW).

**Figure 5:**
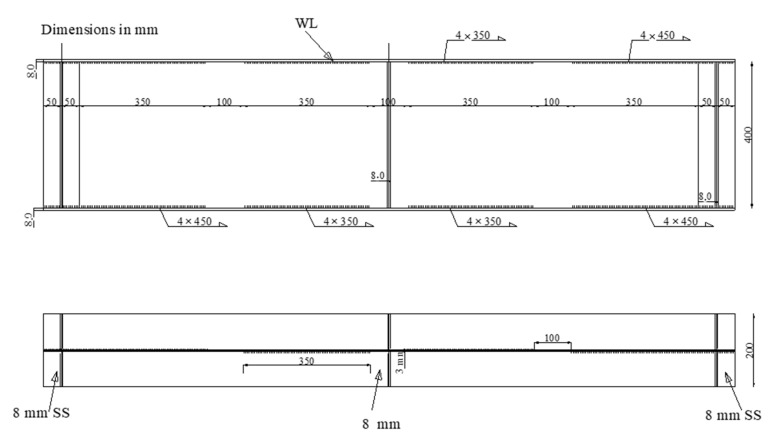
Flat-web specimen-intermittent welding line (FW35WL).

The first and second specimens (CW20IFNW and CW20IFNWFS) had HF lengths of 200 mm. The first specimen (CW20IFNW, Figure 2a) was fabricated without flange transversal stiffeners, whereas the second specimen (CW20IFNWFS, Figure 2b) had flange transversal stiffeners. For these two specimens, the welding was performed between the HF and the flanges only. The third and fourth specimens (CW35IFNW and CW35IFWL, Figure 3) were fabricated using 350-mm-long HFs. The third specimen (CW35IFNW, Figure 3b) was manufactured with welding only between the HF and the flanges, and the fourth specimen (CW35IFWL, Figure 3a) was fabricated with continuous welding between the flanges and the corrugated web. The fifth specimen (CW26IFNW) was fabricated by welding a 260-mm-long HF to the two flanges (Figure 4). The last specimen (FW35WL) was fabricated with a plane steel web. The connection between the flanges and the web consisted of intermittent weld lines (350 mm length) at intervals of 100 mm along the beam length (Figure 5).

### 3.2. Properties of Materials

**Figure 6:**
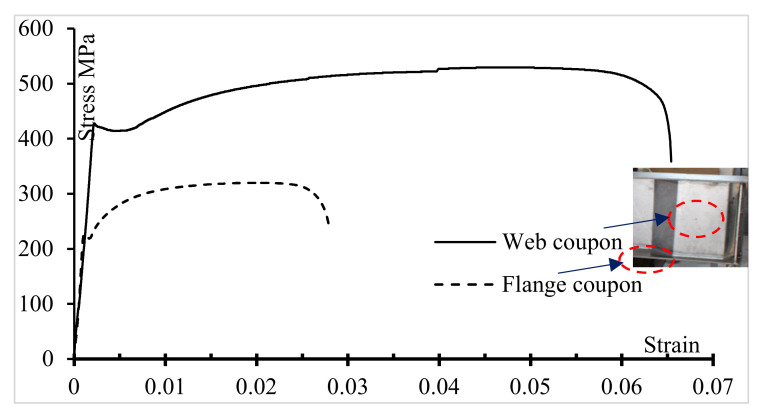
Stress-strain curves—Web and Flange samples.

To determine the mechanical properties of the structural steel used in this study, six standard tension coupons were cut from each specimen: three from the flange and three from the web. The coupons were cut as far as possible from the flame-cut side and machined to the nearest 0.01 mm. The coupons were prepared and tested. All samples were 50 mm wide and 500 mm long, whereas the thickness was according to the corresponding steel member. The tension coupons were tested in a 2000 kN capacity displacement-controlled testing machine using friction grips to apply the loading. The stress-strain curves were consequently plotted, as shown in Figure 6, for the flange and web steels. The material properties, such as the maximum strain, elastic modulus (*E*), and yield and ultimate stresses (*f_y_* and *f_u_*, respectively) obtained from these tests are presented in Table 2.

### 3.3. Test Setup and Instrumentation

**Figure 7:**
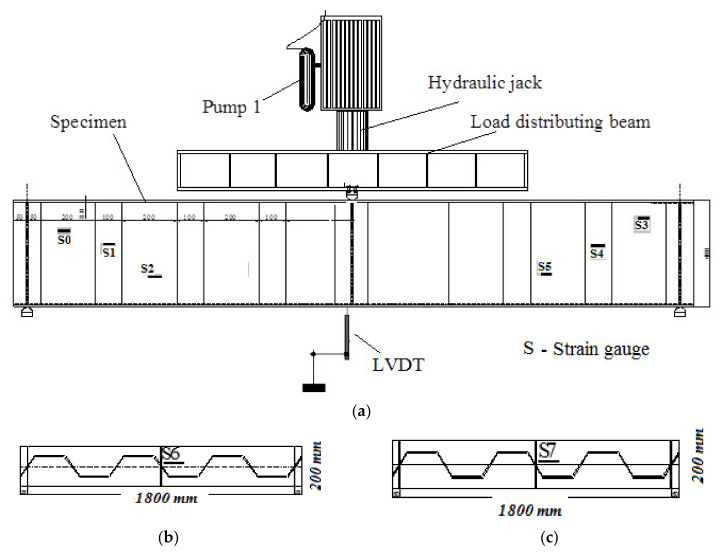
Instrumentations and Test setup: (**a**) Test setup and Locations of flange stiffeners and strain gauges in specimens; (**b**) Gauge in top surface of top steel flange; (**c**) Gauge in bottom surface of bottom steel flange.

The specimens were tested using a ‘2000 kN-capacity’ testing frame. To avoid local flange/web failure, the test specimens were placed in line with the end-bearing stiffeners over the supports at their ends, as shown in Figure 7. The centre of the beam was subjected to a concentrated line load. The unbraced length of the compressive flanges of the specimens was 1800 mm, according to the locations of the supports. The specimens were loaded using a one-line load at the mid-span of the beam with an increment of 0.005 mm/s.

The strains of the specimens were measured using electrical strain gauges with a resistance of 350 Ω, which were attached to the flanges and web, as shown in Figure 7. Six strain gauges—labelled S0–S5—were attached to the web, three from each side of the loading position at intervals of 150 mm along the length direction as shown in Figure 7a. Strain gauge S6 was attached to the top surface of the top flange, as shown in Figure 7b, and strain S7 was attached to the bottom surface of the bottom flange, as shown in Figure 7c. 100-mm linear variable displacement transformers (LVDTs) and dial gauges with an accuracy of 0.01 mm were installed to measure two types of displacement in the specimens: vertical and out-of-plane. To measure the vertical deflections of the specimens, a LVDT was installed under the specimen at the mid-span of the specimen, as illustrated in Figure 7a. Additionally, a dial gauge was installed perpendicular to the length of the top flange at the mid-span of the beam to measure the out-of-plane displacement. All data were recorded using a connected data-acquisition system.

## 4. Test Results and Discussion

### 4.1. Loads Capacities and Failure Mechanisms of Tested Beams

This section discusses the behaviour of the tested specimens with regard to the stiffness, buckling, and failure mode on the basis of the three-point load testing results and test observations. A summary of the test results and failure data (i.e., loads, mode mechanisms, and displacements) is presented in Table 3.

**Figure 8:**
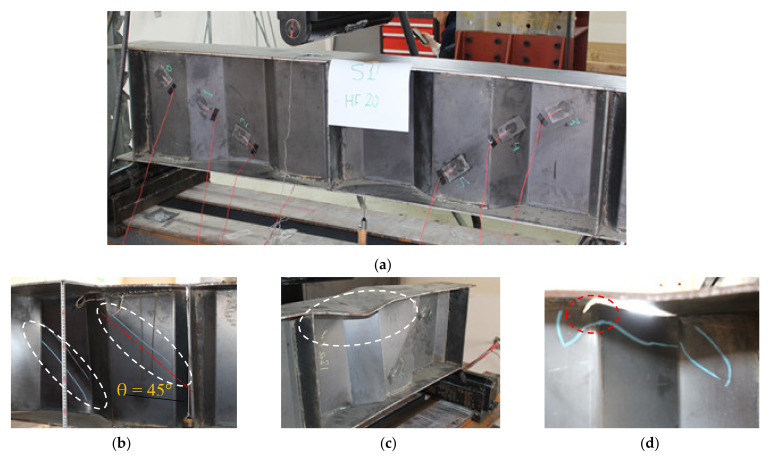
Failure mode of specimen CW20IFNW: (**a**) Specimen CW20IFNW before testing; (**b**) Local web buckling; (**c**) Vertical flange buckling; (**d**) Web tearing.

The three tested steel beams with a corrugated web and without flange stiffeners or continuous welding between the web and flanges (CW20IFNW, CW26IFNW, and CW35IFNW) had similar failure mechanisms (Figure 8). The general failure mechanism comprised three stages: (1) local buckling in the web HF panel (i.e., a downward movement, Figure 8b); (2) vertical flange local buckling (i.e., an upward movement, Figure 8c); and (3) tearing in the web at a certain location (Figure 8d). This mechanism was observed in the three tested beams, regardless of the HF length. In this failure mechanism, it was also observed that local web buckling (WB) and flange buckling (FB) occurred in the panels, close to the loading point. The tearing of the web (WT) appeared in the upper end of the IF at the intersection line with the HF close to the loading line, as shown in Figure 8d. Fundamental and modern theories for shear presume that shear buckling varies between 45° and 60° diagonal tensions. Figure 8b shows the angle of the shear deformation of the HF in the web panel for the CW20IFNW specimen. A previous failure mechanism was also observed for specimen CW20IFNWSF.

**Figure 9:**
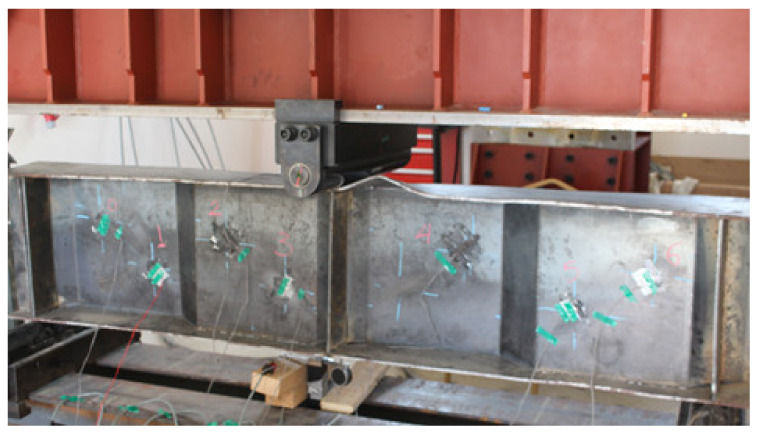
Failure mode of specimen CW35IFWL.

The ultimate load capacities of the three tested beams with HF lengths of 200, 260, and 350 mm were 197, 208, and 217 kN, respectively. The increases in *P_u_* for specimens CW26IFNW and CW35IFNW were approximately 5.6% and 10.2% larger, respectively, than that for specimen CW20IFNW. For specimens CW20IFNW, CW26IFNW, and CW35IFNW, the corrugated web increased *P_u_* by approximately 10.7%, 16.9%, and 20.9%, respectively, compared with the beam with a flat web (FW35WL). These results confirm the effectiveness of the corrugated web for increasing the shear resistance of the beam and delaying the web buckling. The value of *P_u_* for specimen CW20IFNWFS was 225 kN (approximately 14.2% and 26.4% larger than those for specimens CW20IFNW and FW35WL, respectively). Specimen CW35IFWL with continuous welding along the HFs and IFs behaved differently from the previous specimens CW20IFNW, CW26IFNW, and CW35IFNW, as shown in Figure 9.

**Figure 10:**
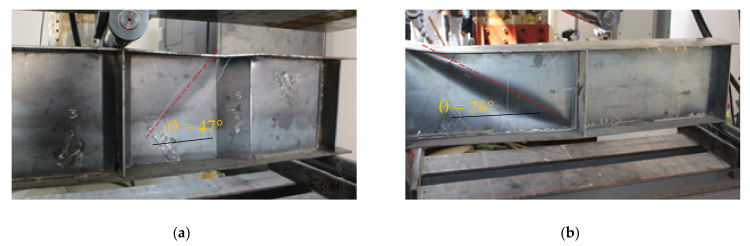
Failure modes: (**a**) Specimen CW35IFNW; (**b**) Specimen FW35WL.

The beam failed owing to flange local buckling only, at a location close to the load line position, with very limited local web buckling in the HF close to the loading position. The ultimate load of beam CW35IFWL was approximately 245 kN (approximately 12.4% and 37.6% larger than those of CW35IFNW and FW35WL, respectively). However, the beam with the flat web (FW35WL) failed owing to global web buckling at a load of 178 kN, as shown in Figure 10b.

Figure 10 also shows a comparison between the web buckling angles of specimens CW35IFNW and FW35WL. The results reveal that the web of specimen CW35IFWL remained intact during the test and contributed to the maintenance of equilibrium until the end of the test. As mentioned previously, the folds were activated after local flange buckling occurred in the HF closest to the load, whereas the remainder of the folds increased the flexural strength for all the corrugated-web specimens. A comparison of the behaviour and capacity between the flat-web and corrugated-web beams revealed that the corrugated-web specimen outperformed its flat-web counterpart with regard to the shear strength and ultimate capacity.

This behaviour was attributed to the superior performance of the corrugation with regard to shear resistance. The experiments conducted by the authors and in other studies revealed that the failure of steel beams subjected to bending and shear is governed by the stress of the web at the maximum shear portions. The results for the tested hybrid SBCW confirmed that as the HF length increased, *P_u_* increased. Moreover, *P_u_* was increased for the beam with continuous welding and flange stiffeners compared with its partially welded counterpart. Further theoretical and numerical investigations are needed to determine the relationships between the HF length and the web aspect ratio and flexural strength for hybrid SBCWs.

### 4.2. Load-Deflection Curves of Tested Beams

**Figure 11:**
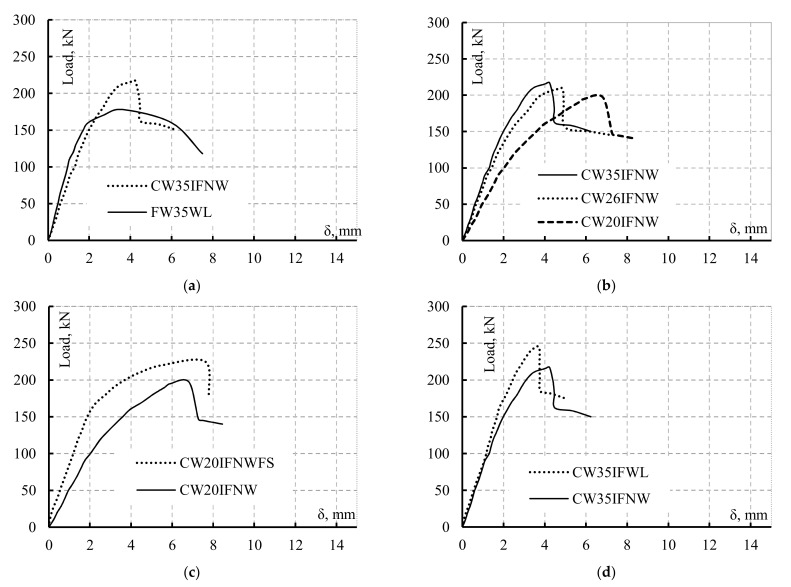
Mid-span vertical deflection vs. vertical loads of the tested beams: (**a**) Effect of the web conditions; (**b**) Effect of the HF length; (**c**) Effect of the stiffeners; (**d**) Effect of the welding conditions.

Figure 11 shows the mid-span vertical deflections of the test specimens. As shown in Figure 11a, comparing the behaviour of the flat-web specimen FW35WL with that of its counterpart CW35IFNW revealed that the flat-web specimen exhibited a high initial stiffness but a low ultimate capacity.

The two tested specimens (CW26IFNW and CW35IFNW) exhibited nearly identical linear behaviour until the deflection reached 1.5 mm, and then local web buckling started to occur, as shown in Figure 11b. This small deflection, which was smaller than L/300, significantly affected the flexural strength of the corrugated-web beams. Additionally, the CW20IFNW specimen exhibited both a low initial stiffness and a low ultimate capacity. Figure 11c presents a comparison between the load-deflection responses of the two specimens with a HF length of 200 mm (CW20IFNW and CW20IFNWFS). The two specimens exhibited different load-deflection responses until the deflection reached 6.3 mm, at which point specimen CW20IFNW reached its ultimate load; CW20IFNWFS reached its maximum load at a deflection value (δ) of approximately 7.8 mm. The specimen with flange stiffeners exhibited a higher initial stiffness and higher ultimate capacity. The results for these two specimens (CW20IFNW and CW20IFNWFS) revealed the significant effects of the flange stiffeners on the stiffness and load capacity of the SBCWs. The two specimens (CW35IFNW and CW35IFWL) exhibited identical linear elastic responses until the deflection reached 1.3 mm; then, the stiffness of beam CW35IFNW decreased. Specimen CW35IFWL exhibited a linear elastic response until δ = 2 mm and reached its *P_u_* at δ = 3.8 mm, whereas specimen CW35IFNW reached its *p_u_* at δ = 4.2 mm (Figure 11d). As shown in Figure 11b,d, the stiffness of the SBCW increased with the HF length or with continuous welding up to *p_u_*.

### 4.3. Efficiency of Transversal Stiffeners

**Figure 12:**
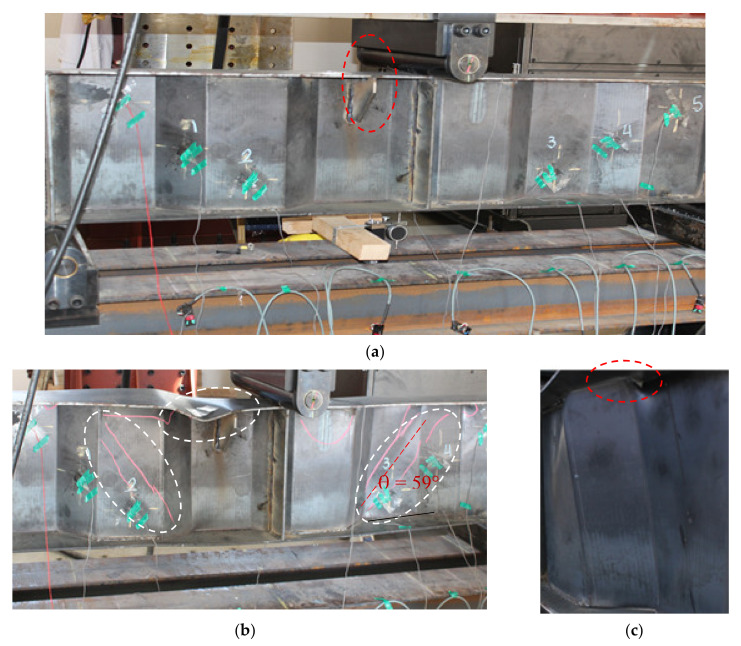
Details and failure mode of specimen CW20IFNWFS: (**a**) Specimen under testing; (**b**) Local web buckling and vertical flange buckling; (**c**) Web tearing.

Beams subjected to flexural loading develop their ultimate strength after flange buckling, depending on their dimensions and yield strength. The bending resistance of corrugated-web beams is controlled by a reduction factor, as stated in the design rule of EN1993-1-5 Annexe D [35]. Theoretical and experimental results indicated that flange local buckling is controlled by the slenderness ratio of the maximum outstanding length of the flange. Adding transversal stiffeners to the top flange in certain positions may increase the rigidity. However, the stiffeners significantly increased the buckling resistances of the flanges, compared with the flanges without stiffeners. The position of the transverse stiffeners is considered to be the most important parameter affecting the buckling behaviour and flexural capacity of SBCWs. The flange with transversal stiffeners outperformed its counterpart with regard to the post-buckling (ultimate) behaviour. The strain measurements indicated that the flanges were not activated effectively until the web buckling. After the web buckling, the strains gradually increased until the top flange buckled. The web buckling rapidly propagated, reducing the integrity of the flange and its ability to withstand compression in the perpendicular direction. The failure was controlled by flange local buckling due to reductions in the outstanding length area of the flange. The failure mechanism of the specimen with transversal flange stiffeners is shown in Figure 12. These results indicate that the transversal flange stiffeners enhanced the post-buckling behaviour of the other tested specimen (CW20IFNW) and increased the ultimate load. It can be concluded that the specimens with flange stiffeners exhibited higher elastic and inelastic responses and enhanced resistance against the bending moment.

Notably, specimen CW20IFNWFS with flange transversal stiffeners exhibited a similar failure mechanism, but local buckling occurred in a different position, as shown in Figure 12b, and the ultimate capacity increased. The CW20IFNWFS failure mechanism was due to web and flange local buckling followed by web tearing, at a location very close to the support.

### 4.4. Load-Strain Curves of Tested Beams

**Figure 13:**
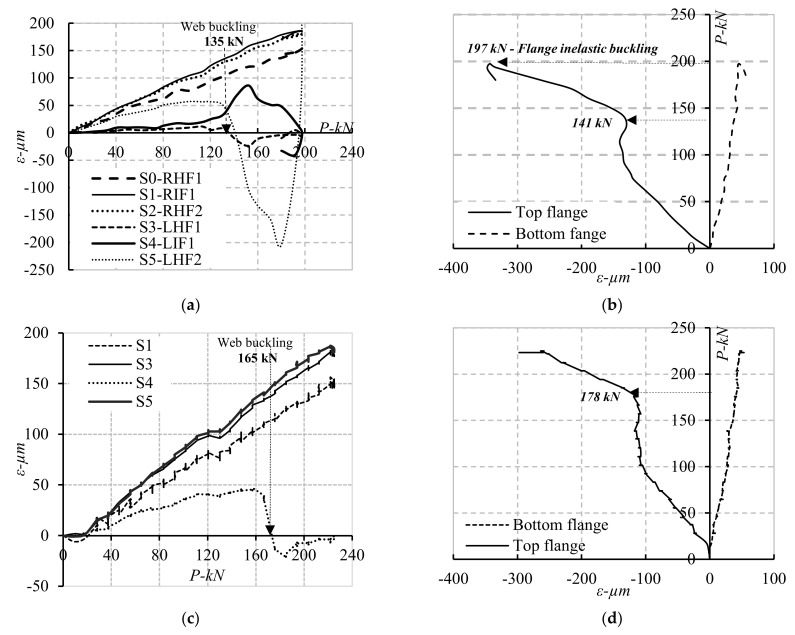
Strain development in the Horizontal and inclined web folds and top and bottom flanges; (**a**) CW20IFNW Web-HZ and IN folds; (**b**) CW20IFNW-Top and bottom flanges; (**c**) CW20IFNWFS Web-HZ and IN folds; (**d**) CW20IFNWFS-Top and Bottom flanges.

To study the elongation behaviour at different positions of the tested specimens, the strain vs. loading curves were plotted for each specimen at different loading stages. Specimens CW20IFNW, CW26IFNW, and CW35IFNW had similar failure mechanisms. This section presents the behaviour of specimen CW20IFNW, which was selected as a sample. Figure 13 shows the measured strains for the horizontal and inclined web folds and flanges (top and bottom). The strain results were almost identical and can be represented by five stages with negligible or minor changes. Figure 13a presents the strains measured at six different positions connected to the web panels of specimen CW20IFNW. For instance, strain gauge S4 indicated that in the first stage, the strain increased linearly with the increasing load until the local web buckling load was reached. The strain then increased while the applied load remained constant. In the third stage, the strain increased linearly with the increasing applied load. In the fourth stage, the strain increased rapidly, while the applied load remained constant. In the last stage, the strain increased linearly in the opposite direction to the applied load until web distortion interrupted the measurement. Furthermore, the strains of the top and bottom flanges for specimens CW20IFNW, CW26IFNW, and CW35IFNW were almost identical, and the sample results are plotted for different loading stages in Figure 13b (specimen CW20IFNW). The local web and flange buckling for the inclined and second HFs were visible for specimen CW20IFNW at loads of 135 and 141 kN, respectively.

Figure 13c,d shows the measured stains for the specimen with flange stiffeners (CW20IFNWFS) in the flanges and web folds at different loading stages. The local web and flange buckling for the IF and second HF were visible for specimen CW20IFNW at loads of 165 and 178 kN, respectively. Figure 13b,d shows the strain responses of the top and bottom flanges, respectively, at the mid-span. The top flange exhibited a variable level of stress; higher levels were observed in the area of maximum outstanding length closer to the loading position. The load-strain curves for both cases (with and without flange stiffeners) exhibited three stages. In the first stage, the strain increased linearly with the increasing load until a certain load was reached, at which point local buckling of the flange started to occur. In the second stage, the strain remained almost constant as the load increased. The strain then increased rapidly with the increasing applied load until the strain could not be measured in the top flange (prior to failure), as shown in Figure 13b,d.

## 5. Theoretical Investigation of Flange Buckling

In this section, two methods are adopted to calculate the bearing capacity of the corrugated web beams and compare with the experimental results. The first method is the bending resistance of corrugated web girders presents by Eurocode 3, where the second method is calculating the inelastic local buckling stress of the flange from the equation proposed by Johnson and Cafolla [36].

### 5.1. EN1993-1-5 Bending Resistance

This subsection presents a numerical calculation of the bending capacity of the CWSB based on design rules stated at EN1993-1-5 Annex D [35]. In this standard, the bending resistance of corrugated web girders should be controlled by a reduction factor, when the girder is also subjected to shear and can be determined by Equation (1).

(1) $$M_{Rd}=min\left[\begin{array}{c} \left(\frac{b_{uf}*t_{uf}*f_{yf,r}}{\gamma_{M0}}*\left(h_{w}+\frac{t_{uf}+t_{lf}}{2}\right)\right);\\ \;\left(\frac{b_{lf}*t_{lf}*f_{yf,r}}{\gamma_{M0}}*\left(h_{w}+\frac{t_{uf}+t_{lf}}{2}\right)\right);\\ \;\left(\frac{b_{uf}*t_{uf}*\chi *f_{yf}}{\gamma_{M0}}*\left(h_{w}+\frac{t_{uf}+t_{lf}}{2}\right)\right) \end{array}\right]$$

where *t_uf_* and *t_lf_* are the upper and lower flange thicknesses, *h_w_* is the web depth, *f_yf_* is the flange yield strength, *ɤ_M_*_0_ is the partial safety factor, and χ is the reduction factor related to the buckling (see details in EN1993-1-5) [35]. When the girder is also subjected to shear, the bending resistance may be reduced by a reduction factor (*f_T_*), which depends on the flange yield strength and the maximum normal stress level coming from transverse bending moment. The reduced bending resistance can be determined by Equations (2) and (3);

(2) $$f_{yf,r}=\;f_{T}\;*f_{yf}$$

**Figure 14:**
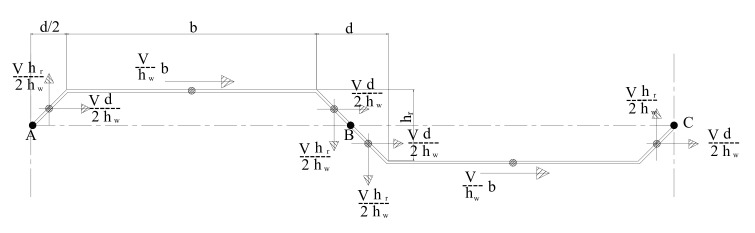
Illustration of the transverse bending moment calculation.

(3) fT=1−0.4∗σx,MzfyfγM0=1−0.4∗6∗Mztf∗bf2fyfγM0

where $f_{yf,r}$ is the value of the yield stress reduced due to transverse moments in the flanges, $\sigma_{x,\left(M_{z}\right)}$ is the stress due to the transverse moment resulting from shear flow in the flanges, $\gamma_{M0}$ is the partial factor. For the corrugation profile shown in Figure 14, the maximum transverse bending moment is calculated as follows:

$$M_{z-max}=M_{B}=\frac{V*h_{r}}{2*h_{w}}\left(b+\frac{3}{4}d\right)+\frac{V*h_{r}*d}{8*h_{w}}+\frac{V*h_{r}*b}{2*h_{w}}+\frac{V*h_{r}*d}{8*h_{w}}-\frac{V*h_{r}*d}{8*h_{w}}$$

Case of welded inclined folds:

(4) $$M_{z-max}=M_{B}=\frac{V*h_{r}}{2*h_{w}}\left(2b+d\right)$$

Case of non-welded inclined folds:

(5) $$M_{z-max}=M_{B}=\frac{V*h_{r}*b}{2*h_{w}}$$

In specimens CW20IFNW and CW35IFNW, the concentrated loads (central load and reactions) located in the middle of the IFs; in this case, Kovesdi et al. [36] stated that Equation (4) was not appropriate for the approximation of stress distribution along the profile length. For beam under three-point load bending, Kovesdi et al. [36] recommended that the additional bending moment can be calculated according to the proposal of Aschinger and Lindner [30] $M_{z}$ (Equation (6)).

(6) $$M_{z}=13\frac{V_{z}}{h_{w}}*d+1.5\frac{V_{z}}{h_{w}}*b*\frac{d}{2}\;\mathrm{Welded}\;\mathrm{IFs}\;\;M_{z}=1.5\frac{V_{z}}{h_{w}}*b*\frac{d}{2}\;\;\;\;\;\;\;\mathrm{Non-welded}\;\mathrm{IFs}$$

(7) $$\bar{\lambda }=\sqrt{\frac{A*f_{y}}{N_{cr}}}=\;\frac{L_{cr}}{i}*\frac{1}{\lambda_{1}}$$

(8) $$\lambda_{1}=\;\pi *\sqrt{\frac{E}{f_{y}}}$$

By applying this equation for specimens with HFs 350 mm and 200 mm, the corresponding stress reduction factors $f_{T}$ were calculated. χ is the reduction factor for the out-of-plane buckling according to the slenderness ratio $\bar{\lambda }$ (6.3 EN1993-1-1), A is the area of the cross-section, $N_{cr}$ is the elastic critical force for the relevant buckling mode, $L_{cr}$ is the buckling length in the buckling plane considered, and i is the radius of gyration about the relevant axis. The results demonstrated that a slenderness ratio of more than 0.2 indicated the reduction factor was equal to 0.7 as well as the partial factor of 1, as stated in [35].

Substituting the dimensions and properties of the tested beams (CW35IFNW and CW35IFWL) into the above sets of equations would determine the equivalent bending resistance of each beam. The ultimate moment resistance, for both beams obtained experimentally, varied between 97.65 kN·m and 110.25 kN·m, which were 1% and 26% higher than the bending resistance calculated from Equation (1), respectively (96.74 kN·m, and 86.85 kN·m respectively). It can be seen that the bending resistance equations of EN 1993-1-5 [35] safely predict the test data of the non-welded inclined fold well and yielded a high safe variation.

### 5.2. Compression Flange Local Buckling Controlled by Flange Properties and Dimensions

To determine the flange slenderness ratio (width-to-thickness), Johnson and Cafolla [37] conducted a study to calculate the capacity of the flange towards local buckling for hybrid SBCW. The largest value of flange overhang (*b_f_* + *h_r_*)/2 was used to determine the flange slenderness by the traditional approach. However, in their study, by considering range of parameters, the authors revealed that the average flange’s overhang, *b_f_*/2, could be used. To determine the inelastic local buckling stress of the flange (*F_fb_*), five tests were conducted [37]. The results of these tests are presented through the following equation:

(9) $$F_{fb}=F_{yf}\left[1-0.88*\left(\lambda_{f}*\sqrt{\frac{F_{yf}}{E}}-0.38\right)\right]\leq F_{yf},$$

where *F_yf_* is the flange yield stress and λ*_f_*is the flange slenderness ratio. The comparison showed that using a flange slenderness ratio of λ*_f_* = (*b_f_*+ *h_r_*)/2*t_f_* provided accurate and slightly conservative results for the flange inelastic local buckling stress (*F_fb_*). Whereas the flange thickness (t*_f_*) and the flange slenderness ratio (λ*_f_*) were too conservative, using *b_f_*/2*t_f_* was potentially not conservative enough.

The analysed beam was considered to reach its ultimate capacity when the outmost steel fibre yielded for the elastic method or when the whole section yielded for the plastic method. The inelastic local buckling stress of the compression flange was calculated from Equation (9) using the properties and dimensions of the fabricated specimens (CW35IFNW and CW35IFWL). The inelastic local buckling stress of the flange, for both beams was constant and equal to 157.93 MPa per Equation (9). By calculating the plastic section modulus, the ultimate moment resistance corresponding to this stress was equal to 95.98 kN·m, and that case could be applied only for the 350 mm horizontal web panel. This value was lower by 1.7% and 15% than the experimental results obtained for specimens CW35IFNW and CW35IFWL, respectively. Equation (9) can safely predict the test result of the welded and non-welded inclined folds specimens, whereas the out-of-plane bending due to corrugation shape neglected in this equation.

## 6. Conclusions

This paper presents the results of laboratory tests of a series of trapezoidal corrugated-web steel beams for investigating the effects of the HF length, welding the IFs between the web and flanges, and transversal flange stiffeners on their behaviour under three-point loading. The test results were discussed, and the failure modes were identified for each beam. Based on obtained results, the following remarks are presented. (i) The observed failure modes indicated that specimens without welded IFs (with or without flange stiffeners) failed in compression because of web and flange local buckling followed by web tearing. The beam with a continuous weld along the HFs and IFs failed owing to flange local buckling only, at a location close to the load line position, with very limited web local buckling in the HFs. (ii) The results for the tested hybrid SBCW confirmed that (in case of the IFs non-welded) as the HF length increased, *P_u_* increased. Moreover, *P_u_* increased for the beam with continuous welding and flange stiffeners compared with its partially welded counterpart. (iii) This study indicated that the effect of non-welded IFs on the ultimate capacity of the SBCW is greater than the effect of fatigue cracks generated along the IFs owing to welding, as reported by Anami et al. [2] and observed for specimens CW35IFNW and CW35IFW. (iv) Flange stiffeners contribute significantly to the overall performance of the SBCW in the elastic and inelastic stages. Additionally, they can positively influence not only the ductile failure mechanism of the SBCW but also the out-of-plane displacement. (v) The flat-web specimen with intermittent welding exhibited a high initial stiffness but a low ultimate capacity compared with its counterpart with a corrugated-web non-welded IF. (vi) EN 1993-1-5 safely predict the test data of the non-welded and welded inclined fold at a safe variation more than that obtained by the proposed equation by Johnson and Cafolla [37]. (vii) The Eurocode 3 and Johnson and Cafolla’s method underestimate the capacity of SBCW, accordingly more theoretical investigation should be conducted to adapt the effect of Flange stiffeners.

## Figures and Tables

**Table 1:** Test matrix and specimen details.

Specimen ID	Web					Flange Dimensions (mm)	Welding between Web and Flanges	Test Variables
	Shape	Dimensions (mm)						
		b	d	h_r_	t			
CW20IFNW	Trapezoidal Corrugated	200	100	100	3	200 × 8	HF only	HF * length
CW26IFNW		260						HF * length
CW35IFNW		350						HF * length
CW20IFNWFS		200						FS
CW35IFWL		350					Both HF * and IF **	WL
FW35WL	Flat	--	--	--	3		intermittent welding line length 35 cm	

* HF and ** IF.

**Table 2:** Elastic moduli, maximum strains, and ultimate and yield stresses.

Coupon Type	Average *f_y_* (N/mm^2^)	Average *f_u_* (N/mm^2^)	Average *E* (Gpa)	Maximum Strain
Flange	225	320	200	0.028
Web	420	530	201	0.065

**Table 3:** Experimental test results.

Specimen	Failure Load (kN)	Failure Mode Mechanisms				Deflection at *p_u_* (mm)	
		Web		Flange	Additional		
		Mode	Angle			δ_out_, mm	δ, mm
CW20IFNW	197	LB *	45°	LB	Web/tear out	35	6.3
CW26IFNW	208	LB	46°	LB	Web/tear out	30	4.9
CW35IFNW	217	LB	47°	LB	Web/tear out	32	4.2
CW20IFNWFS	225	LB	59°	LB	Web/tear out	25	7.8
CW35IFWL	245	--	--	LB	--	23	3.8
FW35WL	178	GB **	26°	--	--	2	7.3

* LB local buckling ** GB global buckling.

## Data Availability

Not included; the data presented in this article is obtained from an experimental study conducted by authors.
